# Phase Field Modeling on By-Product Migration in Crosslinking Polymers for HVDC Cable Insulation Applications

**DOI:** 10.3389/fchem.2022.882347

**Published:** 2022-04-27

**Authors:** Fei Li, Huaqiang Li, Lei Jiang, Dong Wang, Jinghui Gao, Lisheng Zhong

**Affiliations:** ^1^ State Key Laboratory of Electrical Insulation and Power Equipment, Xi’an Jiaotong University, Xi’an, China; ^2^ State Key Laboratory for Mechanical Behavior of Materials, Center of Microstructure Science, Frontier Institute of Science and Technology, Xi’an Jiaotong University, Xi’an, China

**Keywords:** phase field, cross-linked polyethylene, by-product, electric field distribution, HVDC cable

## Abstract

Cross-linking by-products has been considered as one of the crucial factors for dielectric properties of cross-linked polyethylene, which plays an important role in the insulation performance of high voltage direct current (HVDC) cables. It migrates across cable insulation incorporating space charge effect, temperature variation of conductivity, etc., which makes it a long-standing puzzle of manipulating the electric field distribution for HVDC cable insulation, especially with the increasing voltage level. Nevertheless, there still lacks a theoretical model describing the migration of the by-products, especially for cable insulation with sizeable dimensions. In this article, a phase field model is established to simulate the migration of acetophenone (i.e., one of the by-products) through calculating the free energy landscape considering the competition between the diffusion driven by concentration gradient and the uphill diffusion caused by by-product aggregation. The results show that the time-dependence migration during degassing leads to an uneven distribution of acetophenone across the cable insulation, which is in good coincidence with the measured results for a full-size cable. Accordingly, the distortion of the electric field in HVDC cable insulation due to the radial distribution variation of acetophenone has been estimated regarding the relationship between by-product content and conductivity, and a method of manipulating the distribution of acetophenone is proposed to optimize the distribution of the electric field in cable insulation. Our work provides a numerical approach for the simulation of by-product migration in crosslinked polymers for insulation applications.

## 1 Introduction

**FIGURE 1:**
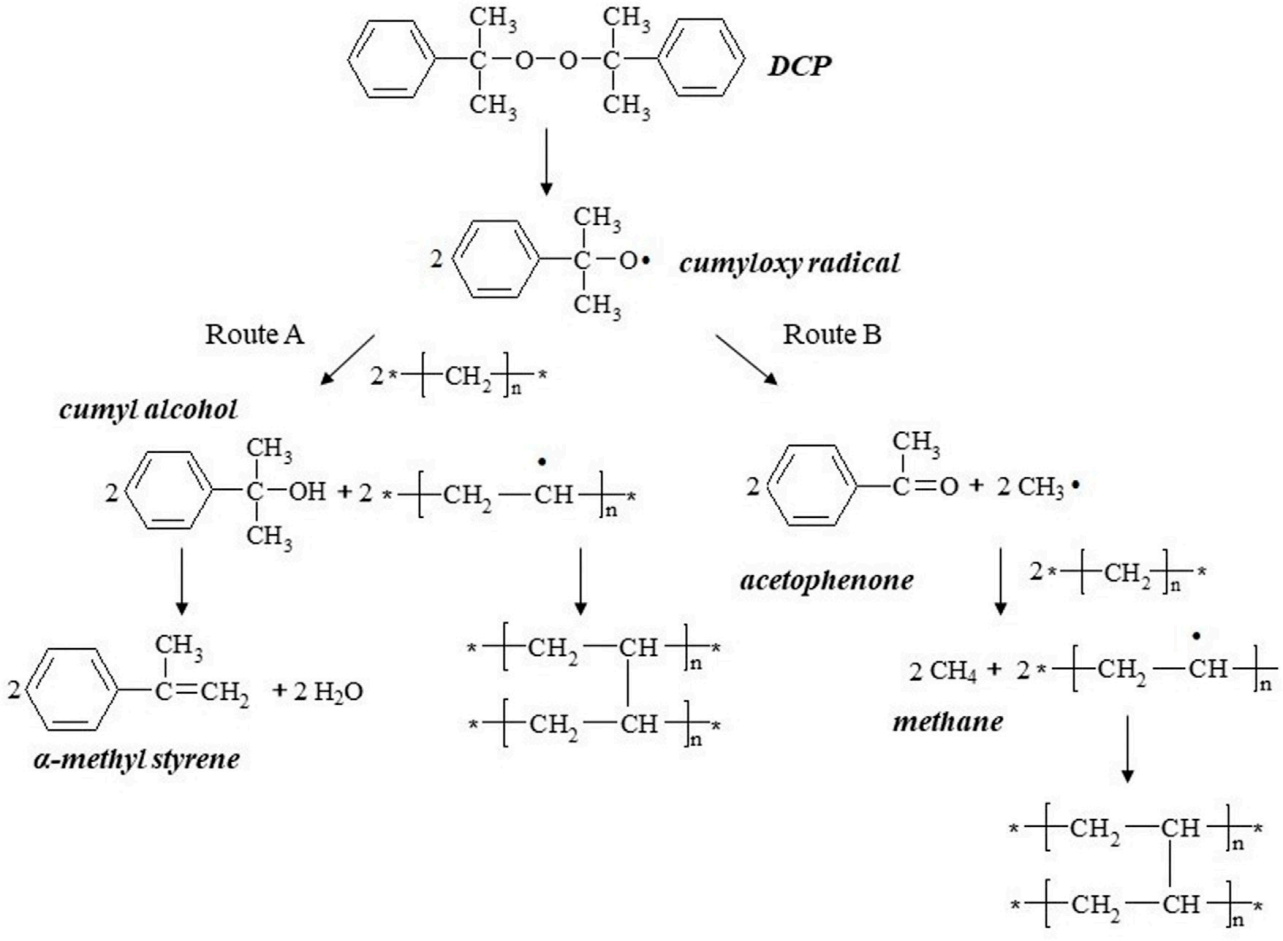
Cross-linking reaction of polyethylene initiated by DCP (Andrews et al., 2006).

To improve the mechanical properties and thermal stability of polyethylene, dicumyl peroxide (DCP) is widely used as the initiator to promote the cross-linking reaction in the production of power cables (Andrews et al., 2006; Sun et al., 2014). The cross-linking reaction in the curing tube is shown in Figure 1. However, the cross-linking by-products, especially acetophenone with high conductivity, largely affect the direct current electrical properties of cross-linked polyethylene (XLPE), and the controlling of by-product content has been considered as one of the long-standing central issues for high voltage direct current (HVDC) cable insulation technology.

Many investigations on the influence of by-products on the electrical properties of the XLPE cable have been conducted. Studies on sheet samples show that the conductivity of polyethylene impregnated with by-products rises with the increase of the by-products’ content (Ohki et al., 2000; Hirai et al., 2001; Hanley et al., 2003; Hirai et al., 2003; and Zhu et al., 2009), while the density of space charge in polyethylene declines with the increase of the by-products’ content (Chong et al., 2006; Lau and Chen, 2006; and Hussin and Chen, 2012), because by-products introduce carriers and promote charge dissipation. Since the degassing process of the full-size cable is more complex than that of sheet samples, the influence of degassing on XLPE cable is also investigated. It is found that the content of by-products in XLPE insulation decrease after degassing, which reduces impurity defects and hetero-charge in XLPE and improves the DC breakdown strength of XLPE (Liu et al., 2017; Tu et al., 2018; Su et al., 2020), whereas the distribution of residual byproducts is radially inhomogeneous after degassing, which means the non-uniform conductivity of the cable insulation (Hjerrild et al., 2002; Vissouvanadin et al., 2011; Sonerud et al., 2014). The results of the electric field simulation indicate that the non-uniform conductivity enlarges the distortion of the electric field in cable insulation and conversely raises the risk of breakdown (Ren et al., 2020; Li et al., 2021). However, these investigations are case studies based on specific cables and lack generality. Therefore, it is of great importance to establish a theoretical model to describe the evolution of by-product distribution with respect to time, electric field, and other external stimuli.

There have been a few works on numerical modeling of the diffusion process of crosslinking by-products. A molecular dynamics (MD) simulation has been employed to study the penetration of small molecules in polyethylene combined with a Monte Carlo (MC) simulation to reflect the diffusion of the by-products (Yang et al., 2019), which has calculated a constrained area with 5 molecule chains, and it is hard to reflect the diffusion in a wide dimension, for example, for full-size cable insulation. On the other hand, an analytical model based on Fick’s law has been established to study the transportation of CH_4_ during the manufacturing of cable (Ruslan et al., 2020; Ruslan et al., 2021), but a complex microstructure for the by-products and its evolution with external stimulus is still lacking.

In this work, the phase field model of acetophenone migration is established based on the Cahn–Hilliard theory to calculate the transportation of acetophenone in a full-size cable insulation during the degassing process. In this model, the conserved field *ϕ*(**x**) is assumed as the order parameter of chemical species concentration, and the free energy density function is used to describe the migration. The radial distribution of acetophenone in the insulation of a 500 kV cable before and after degassing is obtained by using a Fourier infrared spectrometer (FTIR) to check the accuracy of the model. The relationship between acetophenone content and conductivity is investigated to analyze the distortion of the electric field in cable insulation caused by the radial distribution variation of acetophenone. The effects of manipulating acetophenone distribution on electric field distortion in cable insulation are estimated by simulation.

## 2 Experiment and Model

### 2.1 Measurement of Acetophenone Distribution in Cable Insulation

**FIGURE 2:**
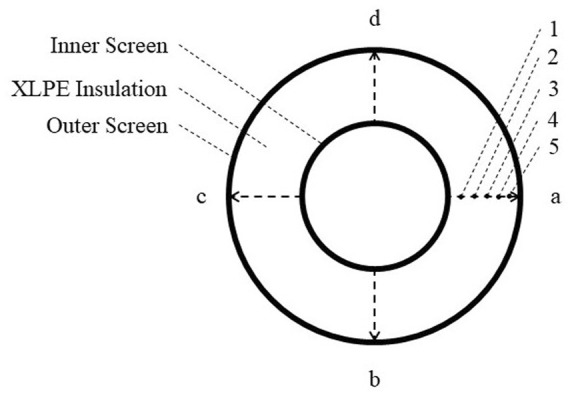
Positions of measuring points on the XLPE sample.

To measure the acetophenone content in cable insulation, the XLPE insulation is selected from an un-degassed 500 kV HVDC cable core, and cut to a sheet sample with the thickness of 0.5 mm along the radial direction. Five measuring points are selected equidistantly in the radial direction of the ring-shaped sample, and the average value of the measuring points in the four directions is taken. The location of the measuring points is shown in Figure 2. After 81 days of degassing at 70°C, the degassed cable insulation is also sliced to measure the content of acetophenone. The semi-quantitative analysis of acetophenone in the samples can be carried out by FTIR based on Bill-Lambert’s law.

To quantitatively analyze the FTIR results, XLPE samples impregnated with acetophenone are prepared. Two XLPE samples with a thickness of 0.5 mm are degassed at 70°C for 48 h in the oven to get rid of all by-products, and immersed into a spectral pure acetophenone solution at 35°C for 48 h to saturation. The relative content of acetophenone in XLPE samples is measured by FTIR every 30 min, and the weight of the samples is measured by a precision balance until acetophenone in the sample is completely volatilized. Then, measure the weight of the blank samples, and calculate the absolute content of acetophenone at each measuring point. Thus, the functional relationship between the relative content and absolute content of acetophenone in the XLPE can be fitted, and the acetophenone absolute content at each measuring point in the slice sample of 500 kV cable core can be calculated.

The relative content of acetophenone in XLPE samples is measured by FTIR with a wave number ranging from 4,000 to 600 cm^−1^. The absorption peak at the wave number of 1,690 cm^−1^ is selected as the characteristic peak of acetophenone. The measured values vary due to the deviation of sample thickness and operating conditions. The absorption peak of methylene - CH_2_ - at 2020 cm^−1^ wave number is the skeleton peak of XLPE, which is immune to other components, and so it is selected as the standard peak to ensure the accuracy of the analysis. The ratio of the height of the absorption peak at 1,690 cm^−1^ to that at 2020 cm^−1^ is the relative content of acetophenone.

### 2.2 DC Conductivity Measurement of XLPE With Different Content of Acetophenone

The cable insulation is peeled on a lathe to obtain sheets with a thickness of 0.2 mm, and the sheets are cut to specimens with a width of 100 × 100 mm. The specimens are degassed in an oven at 70°C for 48 h to completely remove the by-products, and then immersed in a spectral pure acetophenone solution. XLPE samples with different contents of acetophenone are obtained by controlling the immersion time. The relative content of acetophenone in the samples is measured by FTIR, and then the absolute content of acetophenone is calculated.

A three-terminal electrode is used to measure the conductivity of the XLPE specimens. The diameter of the high voltage electrode is 46 mm, the diameter of the measuring electrode is 26 mm, and the outer diameter of the guard electrode is 46 mm with a gap of 4 mm. A 40 kV low ripple DC power supply and a Keithley 6517B electrometer are also used.

The three-electrode system is put in an oven with a constant temperature of 30°C and the conductivity of the samples is measured under electric stresses of 20 kV/mm, 30 kV/mm, and 50 kV/mm, respectively. The current curve is recorded for 10 min, and the current of the last minute is used to calculate the conductivity of the samples. Two samples are measured at every electric stress to take the average value.

### 2.3 Establishment of Acetophenone Migration Phase Field Model

Assume that the phase field *ϕ*(**x**) is the order parameter of acetophenone concentration in the XLPE/acetophenone solution system. Since there is no chemical reaction during degassing, the total amount of acetophenone remains constant and *ϕ*(**x**) is a conserved field. Assume that *n*_A_=(*ϕ*+1)/2 is the concentration of acetophenone, then *ϕ* = 1 corresponds to the state of pure acetophenone, and *ϕ* = -1 corresponds to the state of pure XLPE. When the phase field is so defined, the free energy of acetophenone can be described by the free energy density function in Eq. 1:

$$f\left(\phi \right)=\text{U}{\left(\phi -1\right)}^{2}{\left(\phi +1\right)}^{2}$$ (1)

*ϕ*(**x**) is a conservative field, so:

$$\frac{\partial \phi \left(x\right)}{\partial t}+\nabla J\left(x\right)=0$$ (2)

Where ***J*** =(J_x_, J_y_, and J_z_) is the flux vector of field *ϕ*(**x**).

The reduction of *F,* which is the total free energy of acetophenone, provides the driving force for the evolution of the field, making the system develop towards the direction of lower free energy and gradually tends to be stable. In a conservative field, the total free energy can only be reduced by the redistribution of the field *ϕ*(**x**). Therefore, the field will flow in the form of -δ*F*/δ*ϕ*(**x**), which is the driving force of migration, making the distribution of *ϕ*(**x**) more uniform. Assume that the flux ***J*** is proportional to the gradient of the driving force, i.e.,

$$J=-\text{M}\nabla \mu =-\text{M}\frac{\delta F}{\delta \phi }$$ (3)

Where *M* is the migration coefficient and *μ* is the generalized chemical potential.

It can be obtained from Eq. 2 and Eq. 3:

$$\frac{\partial \phi }{\partial t}=\nabla \cdot \left[\text{M}\nabla \left(\frac{\delta F}{\delta \phi }\right)\right]$$ (4)

A widely used form of the free energy functional *F* is as follows:

$$F\left[\phi \left(x\right)\right]=\int \left[f\left(\phi \left(x\right)\right)+\epsilon |\phi \left(x\right){|}^{2}\right]d^{3}x$$ (5)

Where *f*(*ϕ*) is the bulk contribution to the free energy density, and the second term in the integrand is a correction for the change of the phase field, to increase the interface energy between different phase domains.

It can be obtained from Eq. 4 and Eq. 5:

$$\frac{\partial \phi }{\partial t}=\text{M}\nabla^{2}\left[\frac{\partial f}{\partial \phi }-2\epsilon \nabla^{2}\phi \right]=\text{M}\left[\frac{\partial^{2}f}{\partial \phi^{2}}\nabla^{2}\phi +\frac{\partial^{3}f}{\partial \phi^{3}}{\left(\nabla \phi \right)}^{2}-2\epsilon \nabla^{4}\phi \right]$$ (6)

The variation of acetophenone concentration in cable insulation with the degassing time can be obtained by solving Eq. 1 and Eq. 6.

## 3 Results and Discussion

### 3.1 The Migration Process of Acetophenone

**FIGURE 3:**
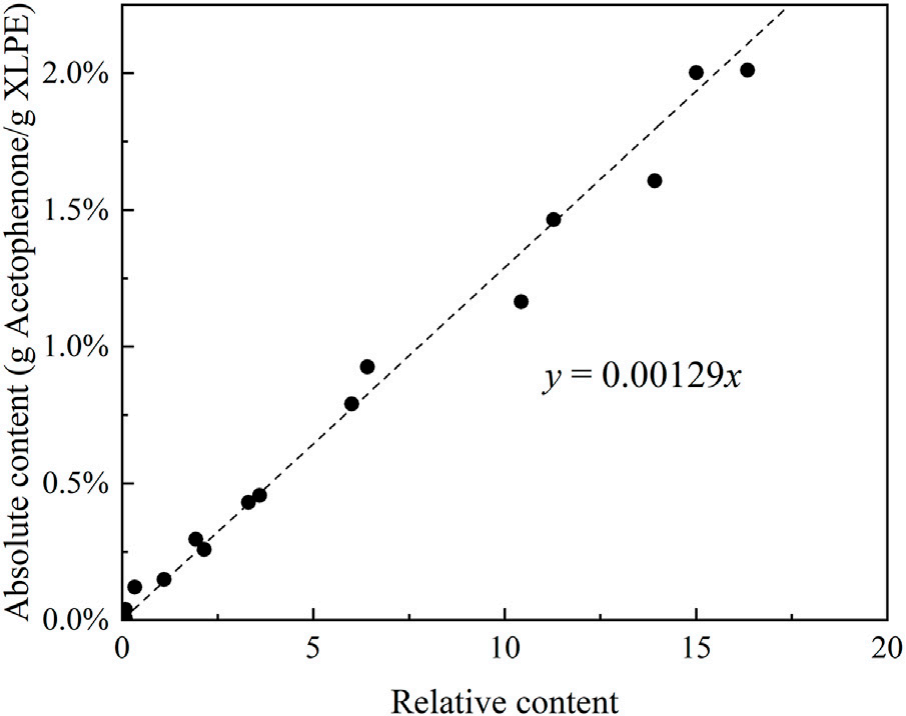
Relative content and absolute content of acetophenone in immersed XLPE samples.

The relative content of acetophenone in immersed XLPE samples is obtained by FTIR, and the absolute content of acetophenone is measured by a precision balance. The measurement results are shown in Figure 3. The analysis indicates that the absolute content of acetophenone has a linear relationship with the relative content of acetophenone, and the functional relationship between them is obtained by fitting.

**FIGURE 4:**
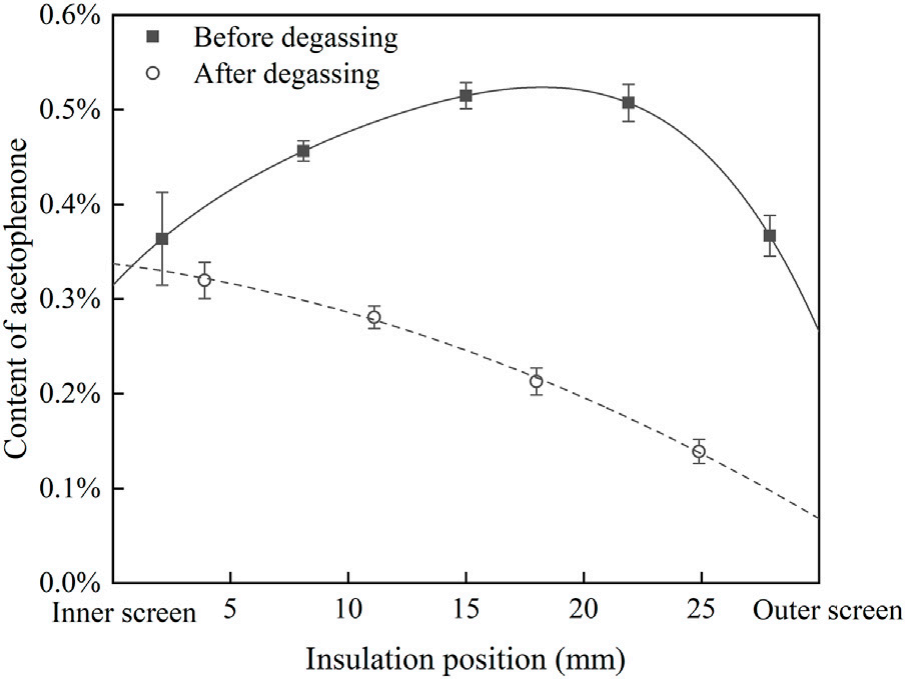
Distribution of acetophenone in cable insulation before and after degassing.

According to the fitting result in Figure 3 and the FTIR results of cable insulation samples, the distribution of acetophenone in a 500 kV HVDC cable insulation before and after degassing is calculated, as shown in Figure 4. Before degassing, the distribution of acetophenone in the insulation is high in the middle and low on both sides. DCP participates in the cross-linking reaction after decomposition, and by-products such as acetophenone and cumyl alcohol will be produced. More cumyl alcohol will be produced when the reaction temperature is low, and the proportion of acetophenone in the by-products will increase with the rise of temperature (Bailey and Godin, 1956; Chodak and Bakos, 1978). In the process of cable production, the cable insulation is heated by high-pressure nitrogen to promote DCP decomposition and initiate the cross-linking reaction. Due to the low thermal conductivity of the insulation layer, the insulation temperature gradually decreases from outside to inside, which makes the higher concentration of acetophenone produced by the cross-linking reaction in the outer insulation. At the same time, the acetophenone in the outer insulation will also migrate to the nitrogen through the outer screen layer, which makes the acetophenone concentration of the outer insulation gradually decrease, and the distribution of acetophenone in the cable insulation finally shows the result of high in the middle and low on both sides. After 81 days of degassing, the distribution of acetophenone in the cable insulation showed a trend of high inside and low outside, indicating that acetophenone migrates from the middle insulation with high concentration to the inner insulation and outer insulation with low concentration. At the same time, acetophenone in the outer insulation migrates into the air through the outer screen layer, while acetophenone in the inner insulation cannot migrate inward due to the obstruction of the copper conductor. It is also found that the migration speed of acetophenone in the XLPE insulation layer is slow. After 81 days of degassing, there was still about 50% acetophenone remaining in the cable insulation, and so it was difficult to completely remove acetophenone from XLPE through degassing.

The migration of acetophenone in cable insulation during degassing will be discussed in the single well model and the double well model.

#### 3.1.1 Single Well Model

The free energy density function of Eq. 1 is a double well function, and the positions of the potential well are φ = ±1, i.e., the content of acetophenone *n*_A_ = 0 and *n*_A_ = 1. However, the maximum content of acetophenone in XLPE is about 2% according to the previous experimental results, which means only a small segment of Eq. 1 is applicable. Thus, the phase field model described by Eq. 1 and Eq. 6 is actually a single potential well model.

**FIGURE 5:**
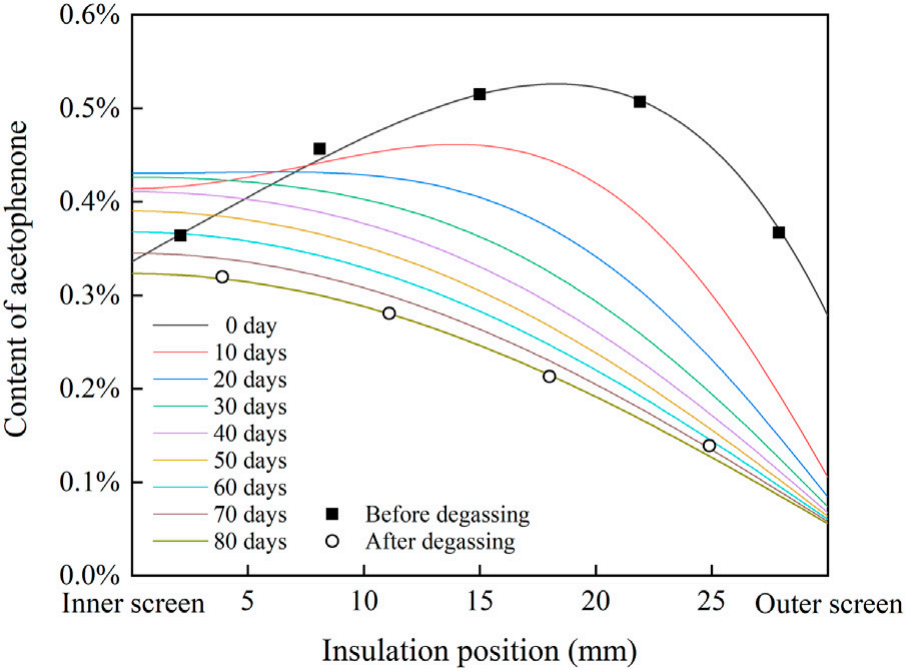
Radial distribution of acetophenone in cable insulation modeling by the phase field in the single well model.

Assume that U = 1; ϵ = 4. Considering that the cable core is wound on the reel for degassing, the acetophenone volatilized from the cable is heavier than the air and cannot be quickly taken away by the airflow, and so we assume that the content of acetophenone in the air on the surface of the cable core *n*_air_ = 0.02%. Because the copper conductor is filled with water blocking glue, acetophenone can hardly pass through, assume that M_conductor_ = 0. Due to a large amount of carbon black in the screen layer, which can adsorb acetophenone, the migration rate of acetophenone in the screen layer is lower than that in the insulation layer, assuming that M_screen_ = 1/3M_insulation_. Set the initial value according to the fitting results of acetophenone content in an un-degassed cable insulation in Figure 4, and substitute the aforementioned parameters into the model for calculation. When M_insulation_ = 0.003, the radial distribution of acetophenone in cable insulation during degassing is shown in Figure 5.

It is found that the distribution curve of acetophenone after 80 days of degassing calculated in the single well model is consistent with the measured results of the degassed cable. The migration process of acetophenone in the single well model is similar to that described by Fick’s law. Acetophenone migrates from the high concentration region to the low concentration region along the direction of concentration gradient. In the first 20 days of the degassing process, the concentration of acetophenone in the middle insulation is the highest, which migrates to the inner insulation and outer insulation. The concentration of acetophenone in the inner insulation reaches the highest value on the 20th day of degassing. After degassing for 20 days, the concentration distribution of acetophenone in the cable insulation shows a trend of high inside and low outside. Then, acetophenone migrates from the inner insulation to the outer insulation. With the increase of degassing time, the concentration gradient of acetophenone in the cable insulation gradually decreases, and so the migration rate slows down and the degassing efficiency decreases.

#### 3.1.2 Double Well Model

Considering the maximum content of acetophenone in XLPE is about 2%, assume that the positions of the potential well are *n*_A_ = 0 and *n*_A_ = 0.02, then the free energy density function of Eq. 1 is changed as follows:

$$f\left(\phi \right)=\text{U}{\left(\phi +0.96\right)}^{2}{\left(\phi +1\right)}^{2}$$ (7)

**FIGURE 6:**
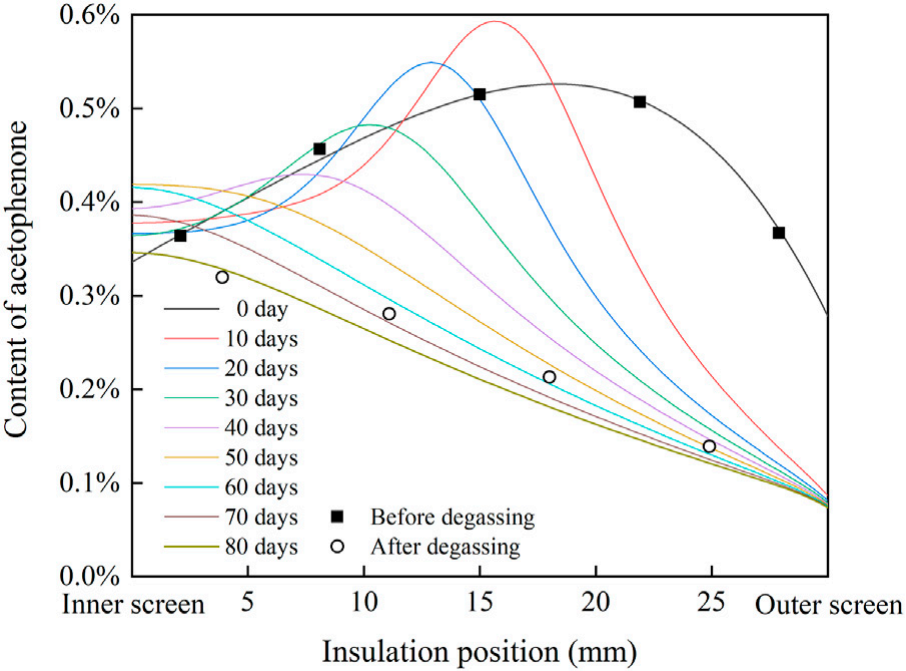
Radial distribution of acetophenone in cable insulation modeling by the phase field in the double well model.

Assume that U = 256; ϵ = 4; *n*_air_ = 0.02%; M_conductor_ = 0; M_screen_ = 1/3M_insulation_. Set the initial value according to the fitting results of acetophenone content in an un-degassed cable insulation in Figure 4, and substitute the aforementioned parameters into the model for calculation. When M_insulation_ = 0.084, the radial distribution of acetophenone in cable insulation during degassing is shown in Figure 6.

The distribution curve of acetophenone after 80 days of degassing calculated in the double well model is also close to the measured results of the degassed cable. Different from the diffusion model based on Fick’s law, the migration process of acetophenone in the double well model shows an obvious uphill diffusion phenomenon. In the first 40 days of the degassing process, acetophenone in the low concentration area of XLPE insulation migrates to the high concentration area. At the same time, because acetophenone volatilizes to the air through the outer screen layer, the content of acetophenone in the outer insulation decreases continuously, which makes the high concentration area move inward and the peak of the concentration curve decrease gradually. After 50 days of degassing, acetophenone migrates from inside to outside along the direction of the concentration gradient. With the increase of degassing time, the degassing efficiency of acetophenone in cable insulation decreases.

The calculated results of the single well model and the double well model are close to the measured results of the degassed cable. The migration process of the double well model shows that there is uphill diffusion of acetophenone during degassing, and an area of high concentration acetophenone appears in cable insulation. However, due to the lack of measured data of acetophenone distribution in the cable insulation during degassing, it is difficult to determine which phase field model is more accurate, and it needs to be further studied.

### 3.2 Influence of Acetophenone on Electric Field Distribution

The analysis indicates that the conductivity of XLPE has an exponential relationship with the field strength and acetophenone concentration at 30°C and 20–50 kV/mm electric stress. Therefore, Eq. 8 is used for fitting:

$$\gamma \left(E,n\right)=\mu \cdot E^{\alpha }\cdot n^{\beta }$$ (8)

where γ is the conductivity of XLPE, S/m; *E* is the electric stress, kV/mm; *n* is the content of acetophenone; *μ* is the constant dependent on material; *α* is the coefficient related to the electric stress; *β* is the coefficient related to the content of acetophenone.

After fitting, *μ* = 1.182 × 10^−11^; *α* = 0.603; *β* = 1.037; correlation coefficient R^2^ = 0.746.

**FIGURE 7:**
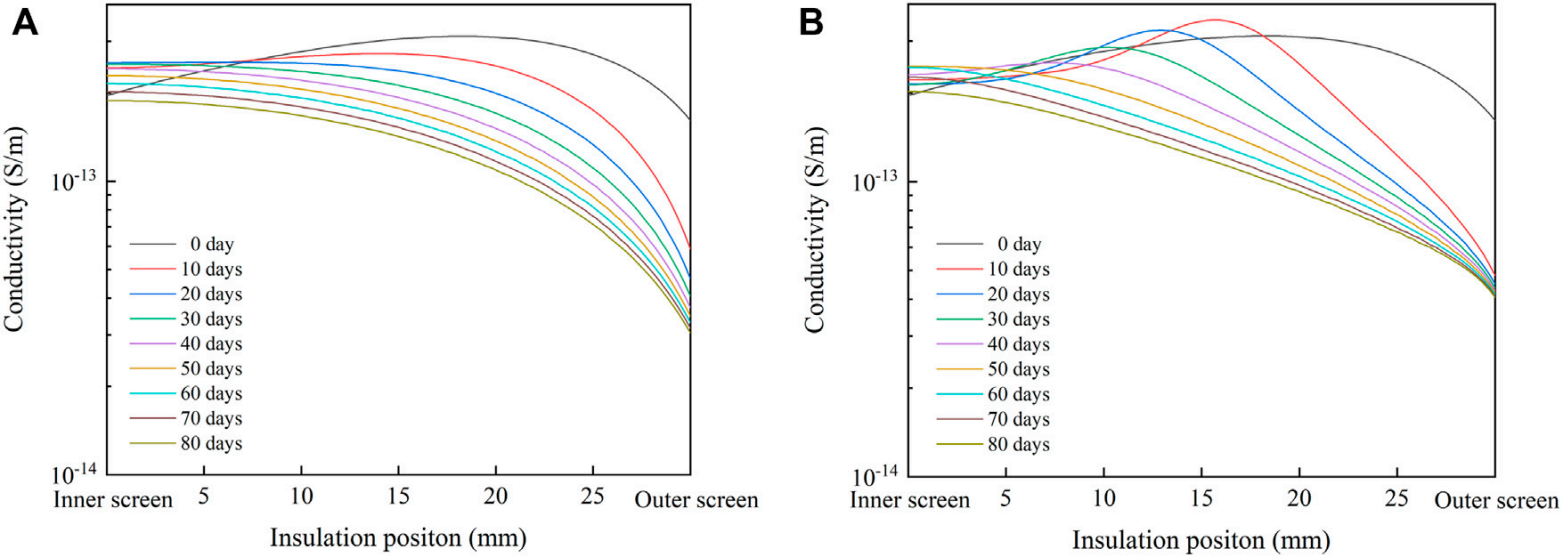
Radial conductivity distribution of cable insulation under 30°C and 20 kV/mm. **(A)** Single well model; **(B)** double well model.

Substituting the aforementioned parameters into the results of Figure 5 and Figure 6, the radial conductivity distribution of cable insulation at 30°C and 20 kV/mm is shown in Figure 7. In the single well model, the radial conductivity distribution of cable insulation changes from high in the middle and low on both sides to high inside and low outside, and the conductivity of insulation decreases gradually with the progress of degassing. In the double well model, the radial conductivity distribution of cable insulation shows a trend of high in the middle and low on both sides until 40 days of degassing, and the peak of conductivity moves to the inner insulation. Moreover, on the 10th and 20th days of degassing, the maximum insulation conductivity is even higher than that of the un-degassed cable, and the radial conductivity distribution changes to the trend of high inside and low outside after 50 days of degassing. In the single well model and the double well model, the change of radial conductivity distribution is similar to that of radial distribution of acetophenone. During degassing, the conductivity of insulation declines obviously with the decrease of acetophenone concentration, but the difference between the conductivity of inner insulation and outer insulation after degassing is close to an order of magnitude, which is significantly higher than that before degassing, which will have a significant impact on the distribution of the electric field.

**FIGURE 8:**
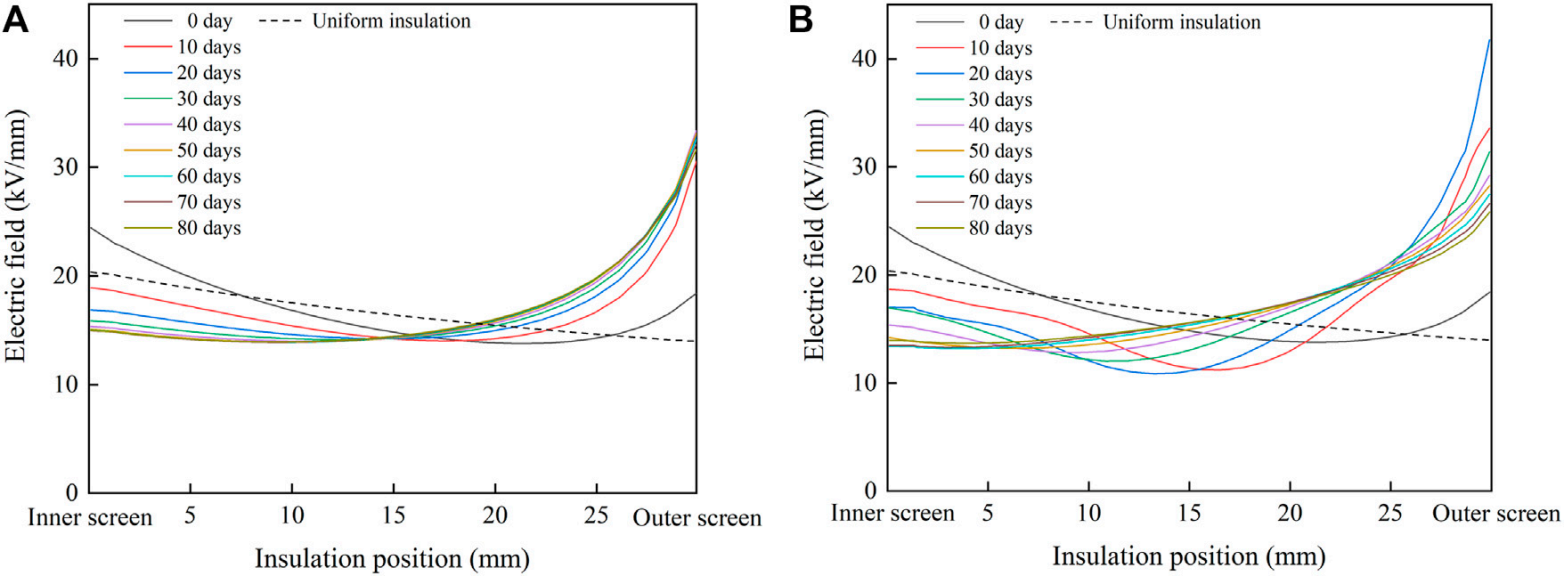
Radial distribution of the electric field in cable insulation. **(A)** Single well model; **(B)** double well model.

The radial distribution of the electric field in the cable insulation under the rated voltage of 500 kV is calculated by finite element simulation software, and the results are shown in Figure 8. When designing cables, XLPE insulation is usually considered as uniform insulation, and the distribution of the electric field in uniform insulation is shown by the dotted line in the figure. The presence of acetophenone distorts the electric field in the un-degassed cable insulation. The electric field of the middle insulation is lower than that of the uniform insulation, while the field strength of the inner insulation and outer insulation is higher than that of the uniform insulation. In single well model, the distribution of the electric field in cable insulation reverses after degassing, and the field strength of inner insulation decreases gradually with the progress of degassing, but the field strength of outer insulation increases rapidly after degassing, which is much higher than the maximum field strength of uniform insulation. After degassing for 30 days, the radial distribution of the electric field does not change significantly. In double well model, the radial distribution of the electric field also reverses after degassing, and the field strength of the inner insulation decreases gradually with the progress of degassing. However, the field strength of the outer insulation increases rapidly after degassing, reaching the maximum value on the 20th day of degassing, and then declines gradually with the progress of degassing. After 80 days of degassing, the distribution of the electric field in single well model and double well model reverse, and the maximum field strength appears in the outermost insulation. However, the change trends of the electric field in cable insulation during degassing in single well model and double well model are different, which is caused by the different change law of radial distribution of acetophenone in the two models.

**FIGURE 9:**
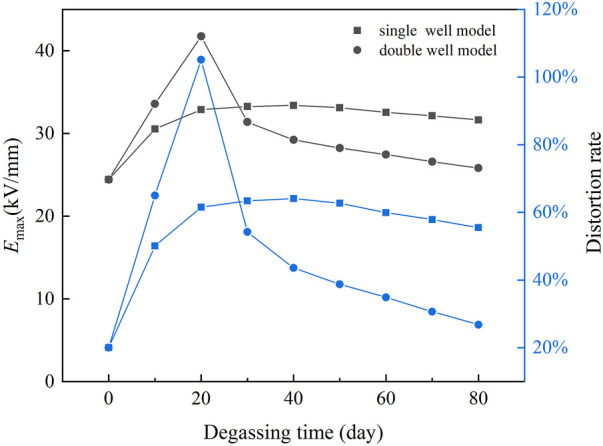
*E*_max_ and distortion rate of the electric field in cable insulation.

The ratio of the maximum field strength of cable insulation exceeding that of uniform insulation is defined as the distortion rate of the electric field in cable insulation. The maximum field strength *E*_max_ and distortion rate are shown in Figure 9. In the single well model, *E*_max_ reaches the maximum value of 33.40 kV/mm on the 40th day of degassing and decreases to 31.65 kV/mm on the 80th day. The distortion rate reaches the maximum value of 64% on the 40th day and decreases to 56% on the 80th day. In the double well model, *E*_max_ reaches the maximum value of 41.74 kV/mm on the 20th day and decreases to 25.81 kV/mm on the 80th day. The distortion rate reaches the maximum value of 105% on the 20th day and decreases to 27% on the 80th day.

After 80 days of degassing, the *E*_max_ and distortion rate in the single well model and the double well model are higher than those before degassing. Instead of improving the radial distribution of the electric field in cable insulation, degassing increases the electric field distortion. In the two models, although the total content of acetophenone in cable insulation decreases, the change of acetophenone distribution also has a significant impact on the conductivity of cable insulation. The conductivity of the inner insulation is much higher than that of the outer insulation, which makes the field strength of the inner insulation decrease and the field strength of the outer insulation increase after degassing. Therefore, the radial distribution of the field strength in cable insulation reverses, and the electric field distortion increases.

### 3.3 Optimizing Electric Field by Manipulating Acetophenone Distribution

According to the analyses in Sections 3.1 and 3.2, the degassing efficiency of acetophenone gradually decreases, and the decline rate of *E*_max_ and distortion rate in cable insulation also slows down with the progress of degassing in both the single well and double well models. It is hard to completely remove acetophenone and improve the electric field distortion in cable insulation by extending the degassing time. Therefore, a method of manipulating the distribution of acetophenone is considered, which means heating the cable at 70°C for 10 days after installing the aluminum sheath on the cable core, to optimize the electric field in cable insulation.

**FIGURE 10:**
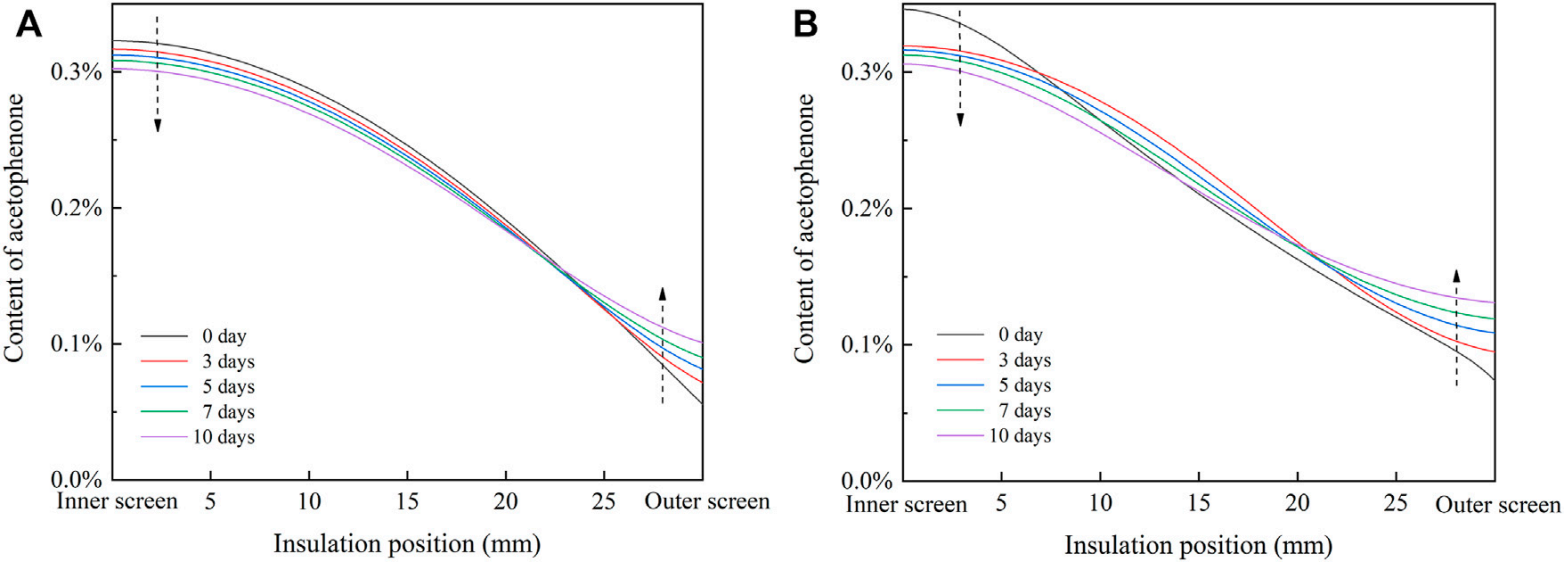
Radial distribution of acetophenone in cable insulation during heat treatment. **(A)** Single well model; **(B)** double well model.

In the process of heat treatment, acetophenone cannot pass through the aluminum sheath and can only migrate in the cable insulation and screen, so M_sheath_ = 0. The radial distribution of acetophenone in cable insulation during heat treatment is shown in Figure 10. In the single well model, a few acetophenones migrate from the insulation to the screen, so the total content of acetophenone in the insulation decreases slightly. The content of acetophenone in the inner insulation and middle insulation decreases, and the content of acetophenone in the outer insulation increases significantly, which reduces the concentration gradient of acetophenone in the insulation. In the double well model, the content of acetophenone in the inner insulation decreases with the progress of heat treatment, the content of acetophenone in the middle insulation increases in the first 3 days of heat treatment and then decreases, and the content of acetophenone in the outer insulation increases gradually, which means the radial distribution of acetophenone in the insulation is more uniform after heat treatment.

**FIGURE 11:**
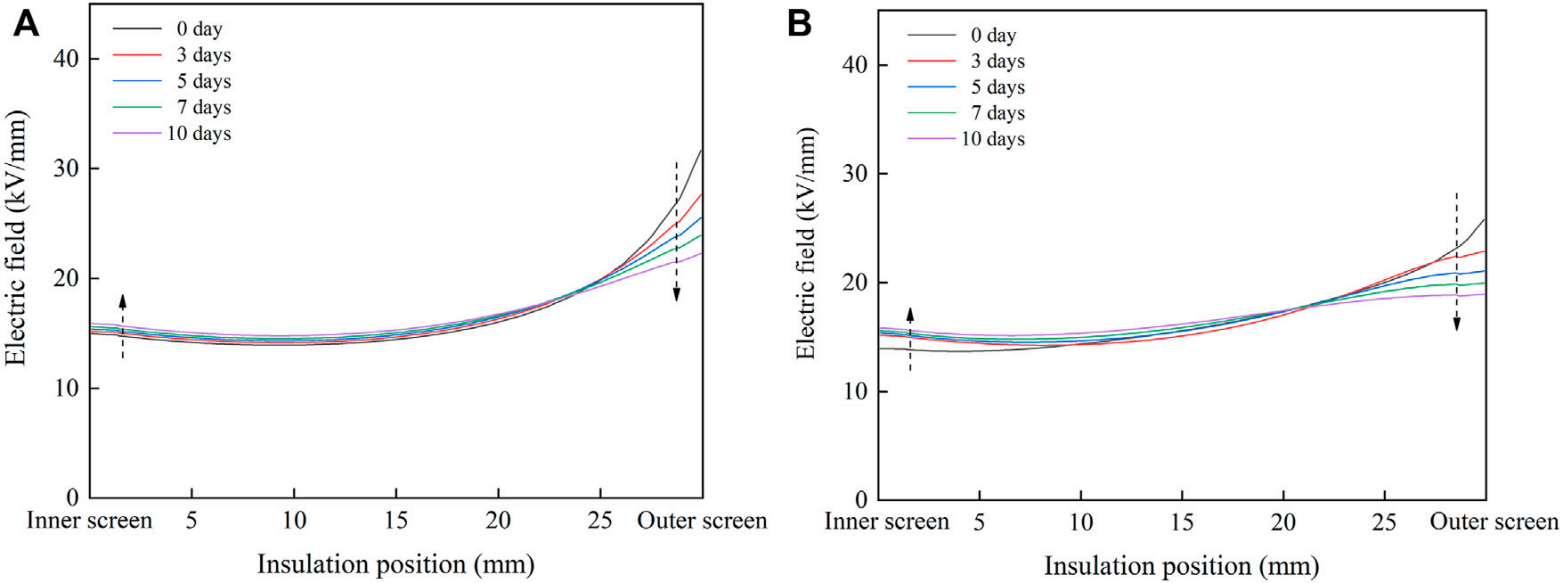
Radial distribution of the electric field in cable insulation during heat treatment. **(A)** Single well model; **(B)** double well model.

The radial electric field distribution in cable insulation under the rated voltage of 500kV is obtained by the finite element simulation, as shown in Figure 11. In single well model and double well model, the electric field strength of the inner insulation and the middle insulation increases slightly, while the electric field strength of the outer insulation declines significantly, so the distribution of the electric field in cable insulation is more uniform. After heat treatment, the concentration gradient of acetophenone in the insulation decreases, so the difference between the conductivity of the inner insulation and the outer insulation decreases, which reduces the distortion of the electric field.

**FIGURE 12:**
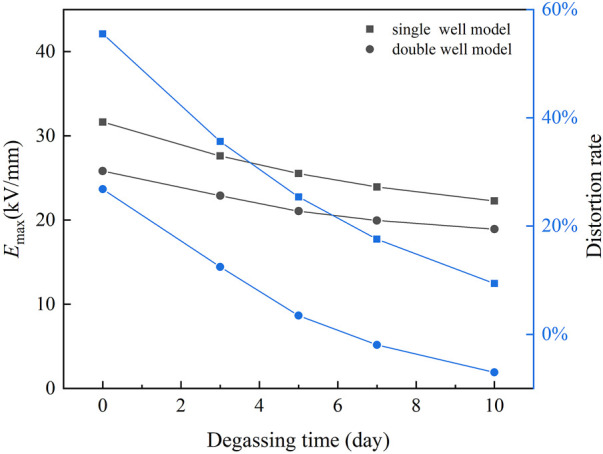
*E*_max_ and distortion rate of the electric field in cable insulation during heat treatment.

In the process of heat treatment, the maximum field strength *E*_max_ and distortion rate of the electric field are shown in Figure 12. In the single well model, *E*_max_ decreases from 31.65 kV/mm to 22.26 kV/mm, and the distortion rate decreases from 56 to 9% after 10 days of heat treatment. In the double well model, *E*_max_ decreases from 25.81 kV/mm to 18.92 kV/mm, and the distortion rate decreases from 27% to - 7%. After 10 days of heat treatment, the *E*_max_ in double well model is even lower than that of uniform insulation. The results of the simulation show that the heat treatment after the aluminum sheath is installed can effectively reduce the distortion of the electric field in the cable insulation and improve the reliability of the cable when it is put into operation.

## 4 Conclusion

In this work, two phase field models of acetophenone migration in full-scale cable insulation are established based on two different free energy density functions. It is found that the simulation results of the two models are in good agreement with the measured results for full-scale cable. In the single well model, acetophenone migrates from the high-concentration area to the low-concentration area along the direction of concentration gradient, while the double well model shows that there is uphill diffusion of acetophenone during degassing. Although the total content of acetophenone in the cable insulation decreases after degassing, the concentration gradient increases, which expands the conductivity difference between inner insulation and outer insulation and increases the electric field distortion. Therefore, a method of manipulating the distribution of acetophenone is proposed to optimize the distribution of the electric field in cable insulation. The simulation results show that although the total content of acetophenone in the insulation is hardly reduced after manipulation, the radial concentration gradient of acetophenone is decreased and the electric field distortion in the insulation is effectively reduced.

## Data Availability

The original contributions presented in the study are included in the article/Supplementary Material, further inquiries can be directed to the corresponding authors.

## References

[B1] Andrews T. Hampton R. N. Smedberg A. Wald D. Waschk V. Weissenberg W. (2006). The Role of Degassing in XLPE Power cable Manufacture. IEEE Electr. Insul. Mag. 22, 5–16. 10.1109/MEI.2006.253416

[B2] Bailey H. C. Godin G. W. (1956). The thermal Decomposition of Dibenzoyl and Di-a-cumyl Peroxides in Cumene. Trans. Faraday Soc. 52, 68–73. 10.1039/TF9565200068

[B3] Chodák I. Bakoš D. (1978). Reactivity of Cumyloxy Radical towards Some Hydrocarbons. Collect. Czech. Chem. Commun. 43 (10), 2574–2577. 10.1135/cccc19782574

[B4] Chong Y. L. Chen G. Hosier I. L. Vaughan A. S. Ho Y. F. F. (2005). Heat Treatment of Cross-Linked Polyethylene and its Effect on Morphology and Space Charge Evolution. IEEE Trans. Dielect. Electr. Insul. 12 (6), 1209–1221. 10.1109/TDEI.2005.1561801

[B5] Hanley T. L. Burford R. P. Fleming R. J. Barber K. W. (2003). A General Review of Polymeric Insulation for Use in HVDC Cables. IEEE Electr. Insul. Mag. 19 (1), 13–24. 10.1109/MEI.2003.1178104

[B6] Hirai N. Minami R. Shibata K. Ohki Y. Okashita M. Maeno T. (2001). Effect of Byproducts of Dicumyl Peroxide on Space Charge Formation in Low-Density Polyethylene. Conf. Electr. Insul. Dielectric Phenomena, 478–483. 10.1109/CEIDP.2001.963585

[B7] Hirai N. Minarni R. Tanaka T. Ohki Y. Okashita M. Maeno T. (2003). Chemical Group in Crosslinking Byproducts Responsible for Charge Trapping in Polyethylene. IEEE Trans. Dielect. Electr. Insul. 10 (2), 320–330. 10.1109/TDEI.2003.1194118

[B8] Hjerrild J. Holboll J. Henriksen M. Boggs S. (2002). Effect of Semicon-Dielectric Interface on Conductivity and Electric Field Distribution. IEEE Trans. Dielect. Electr. Insul. 9 (4), 596–603. 10.1109/TDEI.2002.1024438

[B9] Hussin N. Chen G. (2012). Analysis of Space Charge Formation in LDPE in the Presence of Crosslinking Byproducts. IEEE Trans. Dielect. Electr. Insul. 19 (1), 126–133. 10.1109/TDEI.2012.6148510

[B10] Lau W. S. Chen G. (2006). Simultaneous Space Charge and Conduction Current Measurements in Solid Dielectrics under High Dc Electric Field. Conf. Condition Monit. Diagn., 683–688.

[B11] Li F. Zhong L. Gao J. Li W. Wang C. Tao H. (2021). Electric Field Distribution Considering the Byproducts Inhomogeneity of Crosslinking Insulation in HVDC Cable,” in International Conference on the Properties and Applications of Dielectric Materials, Johor Bahru, Malaysia, July 11–15th, 2021, pp. 318–321. 10.1109/ICPADM49635.2021.9493889

[B12] Liu X. Yu Q. Liu M. Li Y. Zhong L. Fu M. (2017). DC Electrical Breakdown Dependence on the Radial Position of Specimens within HVDC XLPE cable Insulation. IEEE Trans. Dielect. Electr. Insul. 24 (3), 1476–1484. 10.1109/TDEI.2017.006200

[B13] Ohki Y. Hirai N. Kobayashi K. Minami R. Okashita M. Maeno T. (2000). Effects of Byproducts of Crosslinking Agent on Space Charge Formation in Polyethylene-Comparison between Acetophenone and α-methylstyrene. Conf. Electr. Insul. Dielectric Phenomena 2, 535–538. 10.1109/CEIDP.2000.884016

[B14] Ren H. Zhong L. Yang X. Li W. Gao J. Yu Q. (2020). Electric Field Distribution Based on Radial Nonuniform Conductivity in HVDC XLPE cable Insulation. IEEE Trans. Dielect. Electr. Insul. 27 (1), 121–127. 10.1109/TDEI.2019.008345

[B15] Ruslan M. F. A. C. Youn D. J. Aarons R. Sun Y. Sun S. (2021). Numerical Analysis of a Continuous Vulcanization Line to Enhance CH4 Reduction in XLPE-Insulated Cables. Materials 14, 1018. 10.3390/ma14041018 33670006 PMC7926779

[B16] Ruslan M. F. A. C. Youn D. J. Aarons R. Sun Y. Sun S. (2020). Numerical Study of CH4 Generation and Transport in XLPE-Insulated Cables in Continuous Vulcanization. Materials 13, 2978. 10.3390/ma13132978 PMC7372417 32635300

[B17] Sonerud B. Nilsson S. Josefsson S. Huuva R. (2014). The Influence of Degassing and Heat Treatment on the Electric Field Distribution in Extruded HVDC Cables. Electr. Insul. Conf. 1, 29–32. 10.1109/EIC.2014.6869340

[B18] Su P. Wu J. Zhu X. Chen M. Bao Q. Yin Y. (2020). Effect of Degassing Treatment on Breakdown Strength in Direct Current Cable Insulation. Proc. CSEE 40 (2), 663–671. 10.13334/j.0258-8013.pcsee.190833

[B19] Tu Y. Zhang H. Gao Z. Jing Q. Wang Y. (2018). Effects of Degassing Treatment on Crosslinking Byproducts and Space Charge Characteristics in Cross-Linked Polyethylene. Insulating Mater. 51 (4), 58–63. 10.16790/j.cnki.1009-9239.im.2018.04.012

[B20] Vissouvanadin B. Roy S. L. Teyssedre G. Laurent C. Denizet I. Mammeri M. (2011). Impact of Concentration Gradient of Polarizable Species on the Electric Field Distribution in Polymeric Insulating Material for HVDC cable. IEEE Trans. Dielect. Electr. Insul. 18 (3), 833–839. 10.1109/TDEI.2011.5931072

[B21] Sun Y. Person T. J. Jie Ji J. Changkan Zheng C. (2014). Effectiveness of Crosslinking Byproduct Removal from Extruded Power cable. Shenzhen, China: China International Conference on Electricity Distribution, 1739–1743. 10.1109/CICED.2014.6992000

[B22] Yang Y. Narayanan Nair A. K. Sun S. Sun Y. Van Dun J. Kjellqvist J. (2019). Studies of Diffusion of Byproducts Formed by the Peroxide-Induced Cross-Linking of Polyethylene. Int. Conf. Insulated Power Cable B2.4.

[B23] Zhu X. Du B. Gao Y. Ma Z. (2009). Effects of Cross-Linking Temperature on Decay Behavior of XLPE Charge Accumulation. High Voltage Eng. 35 (12), 3154–3158. 10.13336/j.1003-6520.hve.2009.12.003

